# Association between 29 food groups of diet quality questionnaire and perceived stress in Chinese adults: a prospective study from China health and nutrition survey

**DOI:** 10.1186/s12889-024-19308-w

**Published:** 2024-07-09

**Authors:** Jia Zhou, Huan Wang, Christine Pao, Jingjing Zhou, Zhiyong Zou

**Affiliations:** 1 Beijing Key Laboratory of Mental Disorders, National Clinical Research Center for Mental Disorders & National Center for Mental Disorders, Beijing Anding Hospital, Capital Medical University, Beijing, China; 2 Present Address: Advanced Innovation Center for Human Brain Protection, Capital Medical University, Beijing, China; 3 Institute of Child and Adolescent Health, Peking University School of Public Health/National Health Commission Key Laboratory of Reproductive Health, the People’s Republic of China, Beijing, China; 4 Mental Health and Behavioral Science Service, Bruce W. Carter VA Medical Center, Miami, FL USA

**Keywords:** Food groups, Diet quality questionnaire, Perceived stress

## Abstract

**Purpose:**

Diet plays a fundamental role in promoting resilience against stress-related disorders. We aimed to examine the overall and sex-specific association between food groups and perceived stress in adults.

**Methods:**

We analyzed the prospective data of 7,434 adults who completed both the 2011 and 2015 surveys of the China Health and Nutrition Survey (CHNS). The Diet Quality Questionnaire (DQQ) was used to code all the food items of 2011 dietary intake into 29 food groups, and perceived stress in 2015 was measured using a 14-item perceived stress scale (PSS-14). Univariate analysis and logistic regression models were used to examine the relationship between food groups and perceived stress.

**Results:**

People who perceived a higher level of stress (PSS-14 total score > 25) made up 41.5% and 45.1% of the male and female groups, respectively (χ^2^ = 9.605, *p* = 0.002). Individuals with increased intake of food groups such as legumes, other vegetables, other fruits, yogurt, poultry, fish & seafood, fluid milk, and fruit juice were less likely to experience a higher level of psychological stress (OR range: 0.544–0.892, *p* < 0.05). Additionally, we found sex-specific associations between food groups and perceived stress. The difference in the proportion of food groups, such as fluid milk and fish & seafood, between the two stress groups in men was statistically significant (*p* < 0.025). In the female group, the distribution of eight food groups, like legumes and nuts & seeds, between the two stress groups was statistically significant (*p* < 0.025).

**Conclusion:**

This study indicated that food groups were differentially associated with perceived stress.

**Supplementary Information:**

The online version contains supplementary material available at 10.1186/s12889-024-19308-w.

## Introduction

Stress-related disorder is a global problem, and it is estimated that 322 million people live with depression worldwide [1]. Perceived stress, which takes into account the perceived controllability, manageability, and personal impact of situations, has a strong and consistent association with concurrent and subsequent depression and potentially other mental health problems [2]. A previous study by Leng et al. suggested that a higher level of stress can be considered harmful and has a certain degree of negative impact on a person’s health [3]. It impairs emotional, physical, cognitive, and social functioning [4] and puts people at increased risk of stress-related health problems, especially depression [5]. Bremner et al., reviewed the associations between diet, stress, and stress-related disorders. They found that diet can affect mood through direct effects, and stress could lead to stress-related mental disorders, such as depression and posttraumatic stress disorder (PTSD) [6]. In today’s society, we thrive on performance, competition, and perfection, which leads to an insidious increase in stress. Meanwhile, rapidly growing urbanization and the globalization of the food industry have prompted profound shifts away from traditional dietary patterns. Although stress-related disorders (e.g., depression) seems to be heterogeneous disorders with no established mechanism [7], emerging and compelling evidence suggests that diet has a fundamental role in promoting resilience against these disorders [8–10]. A study conducted in China indicated that dietary diversity was found to be inversely associated with psychological stress [11]. Another study conducted in China also indicated that the dietary affected the degree of stress significantly [12]. Wu et al. reported that following a traditional Chinese dietary pattern was associated with a lower risk of depressive symptoms [13].

In terms of the potential mechanism, the structure and function of the brain is dependent upon energy, amino acids, fats, vitamins, minerals, and trace elements provided by food. The immune system [14], inflammatory system [15], antioxidant defense system [16], gut microbiota [17] and neurotrophic factors [18], which moderate the risk for stress-related disorders, operate with the support of nutrient cofactors and phytochemicals. Furthermore, there are two main types of neurotransmitters that affect mood: serotonin, which mainly affects mood, and epinephrine, which affects motivation [19]. Some nutrients in food are the precursors of these neurotransmitters [20], when the body ingests these nutrients, through body processing, the corresponding neurotransmitters can be formed, a certain amount of nutrients can produce a certain amount of neurotransmitters, thereby affecting their concentration levels in the body, and ultimately affecting our mood [21]. For example, the study of Radavelli-Bagatini et al. indicated that fruit and vegetable intake is inversely associated with perceived stress across the adult lifespan [22]. So, although the reality is quite complicated, in theory the emotions or stress can be regulated by adjusting the recipe.

Different food groups may lead to different stress levels. Various studies have illustrated the association between different nutritional factors and psychological health [23], including single nutrients such as magnesium [24], vitamin C [25], B vitamins [26], and omega-3 fatty acids [27], as well as dietary patterns [28] such as ketogenic diet [29], MIND diet, DASH (Dietary Approaches to Stop Hypertension) [30] diet, and the Mediterranean diet [31, 32], and also food groups such as fruits and vegetables [33] and fish [34]. Furthermore, previous studies found that a diet enriched with curcumin promotes resilience against chronic social defeat stress [35]. The latest research reported that psychobiotic dietary intervention has a promising role in reducing perceived stress [36]. People with lower adherence to the Mediterranean diet demonstrated higher perceived stress [37]. These previous findings have shown that diet has a fundamental influence on the perceived stress. Even though relationship between diet and stress can be bidirectional, as a systematic review indicated that stress can lead to disruption to normal eating behaviors [38]. Diet hold promise as potential approaches to address stress-related disorders through future dietary guidelines. Furthermore, although research has shown that females are more vulnerable to the development of depression and other stress related mental disorder [39], studies examining sex-specific association between diet and perceived stress are very limited.

A potential source of variation in the diet-pressure association may be biological sex. For example, sex-based differences was found in the association between dietary fiber intake and the prevalence of very severe stress in men and women [40]. Sex differences were also reported in the relationship between dietary pattern adherence and cognitive function [41]. Lee and Allen found the sex differences in the effect of fruit consumption on depression [42]. A greater understanding of sex-based differences in the association between dietary intake and psychological status would be useful in optimizing existing nutritional interventions and facilitating the execution of new comprehensive interventions intended to enhance the nutritional status and health profile of populations [43].

The Diet Quality Questionnaire (DQQ) was developed by Anna W. Herforth with a global framework of 29 food groups, which aimed to make food groups comparable globally [44]. The DQQ for China with the same 29 food groups was previously developed and evaluated by our research group [45], and we have also previously shown that dietary diversity was inversely associated with stress [11]. However, limited studies have comprehensively investigated the relationship between food groups and perceived stress in a general population in China. Thus, the aim of the current study is to examine the overall and sex-specific associations between 29 food groups from the DQQ for China and perceived stress, using prospective data from the China Health and Nutrition Survey (CHNS). We hypothesized that different food groups lead to different stress levels and that this relationship varies by gender.

## Materials and methods

### Data resource and study participants

The CHNS was an ongoing open-cohort study jointly conducted by the Carolina Population Center at the University of North Carolina and the National Institute of Nutrition at the Chinese Center for Disease Control and Prevention. The project began in 1989 and comprised a total of 10 waves (in 1989, 1991, 1993, 1997, 2000, 2004, 2006, 2009, 2011, and 2015). It used a multistage random-cluster sampling process, including nine provinces (Liaoning, Heilongjiang, Jiangsu, Shandong, Henan, Guangxi, and Guizhou) and three municipalities (Beijing, Shanghai, and Chongqing) that differ in economic development, geography, health indicators, and public resources. CHNS was reviewed and approved by the corresponding institutional review committees (2015,017). Details about the study design are available elsewhere [46].

The present analysis selected Chinese adults who participated in the two rounds of surveys in 2011 and 2015 as longitudinal tracking subjects. In the 2015 wave, the 14-item perceived stress scale (PSS-14) was incorporated into the project for the first time [47]. A total of 15,725 people participated in the 2011 wave. Those excluded from analysis included 2,628 participants aged ≤ 17 in 2015, 4,360 participants lost to follow-up, and 1,303 participants with no/incomplete PSS-14 information. Ultimately, 7,434 participants with information comprised of basic demographic characteristics (i.e., age, sex, weight (kg), height (m), location, province, marital status, and urbanicity index), complete PSS-14 score, and diet information were included in this analysis. The determination process of participants was consistent with our previous article [11].

### Study outcome and other definitions

The PSS-14 is a validated questionnaire developed by Cohen et al. [48], and its Chinese version has been validated [49]. It aims to measure the degree to which situations in one’s life are appraised as stressful, and the items are designed to measure the extent to which one’s life is perceived as unpredictable, uncontrollable, and overloading [48]. The questions were examined on a 5-point Likert-type scale, ranging from 0 = “never” to 4 = “very often”. Scores are obtained by reverse scoring the positively stated items (4, 5, 6, 7, 9, 10, and 13). The total score (range 0–56) was calculated by summing the scores of all 14 items. A higher PSS-14 score indicates a higher degree of perceived stress. There is a lack of studies proposing a standard cut-off score to diagnose or grade stress for PSS-14 *(*Wang et al. [50]. Previous studies have often used medians or quartiles to define cut-off score. In this study, the participants were characterized into two groups based on the median of PSS-14. High reliability was demonstrated in our sample (Cronbach α = 0.83).

### Dietary assessment and food group collection

The dietary information in 2011 was collected by trained nutritionists. They interviewed the participants in their households using 24-hour dietary recall, which is a validated method [51]. Further information on the dietary interview has been described elsewhere [52]. The Diet Quality Questionnaire (DQQ) is a valid and low-burden tool to collect data about the most common food groups consumed by the general population, using sentinel foods (defined as the foods in each food group that were consumed by more than 95% of people) to capture food group level data and reflect healthy dietary patterns [53]. The Chinese version of the DQQ has been adapted and verified [45]. The DQQ can be used to code diet into 29 food groups and it has been adapted to represent foods in the Chinese context [45]. The 29 food groups are included as follows: (1) staple foods made from grains; (2) whole grains; (3) white root/tubers; (4) legumes; (5) vitamin -rich orange vegetables; (6) dark green leafy vegetables; (7) other vegetables; (8) vitamin A-rich fruits; (9) citrus; (10) other fruits; (11) grain-based sweets; (12) other sweets; (13) eggs; (14) cheese; (15) yogurt; (16) processed meats; (17) unprocessed red meat (ruminant); (18) unprocessed red meat (nonruminant); (19) poultry; (20) fish & seafood; (21) nuts & seeds; (22) packaged ultra-processed salty snacks; (23) instant noodles; (24) deep fried foods; (25) fluid milk; (26) sweetened tea/coffee/milk drinks; (27) fruit juice; (28) sugar-sweetened beverages (SSBs) (sodas); and (29) fast food. All foods were grouped into the 29 globally unified food groups with reference to the International Dietary Quality Framework [45, 54]. If the intake of each group of food is greater than 15 g/day, it is judged that the food intake of this group is yes, and vice versa is no.

### Measurements and calculation of covariates

Sociodemographic factors were assessed such as age, sex, weight and height, marital status, and urbanization index. Body mass index (BMI, kg/m^2^) calculated with weight (kg)/[height (m)]^2^ was categorized into four groups: underweight (BMI < 18.5 kg/m^2^), normal (BMI ≥ 18.5 and < 24.0 kg/m^2^), overweight (BMI ≥ 24 kg/m^2^ and < 28 kg/m^2^), and obese (BMI ≥ 28.0 kg/m^2^).

### Statistical analysis

Sociodemographic information is summarized as the mean (standard deviation) for continuous variables and number (percentages) for categorical variables. To determine significant differences between two perceived stress levels (PSS-14 ≤ 25 vs. PSS-14 > 25), χ^2^ tests and Wilcoxon rank tests were used for categorical variables and continuous variables, respectively.

First, univariate analysis was used to analyze the difference in food group consumption between the perceived stress levels. Statistical significance between the two stress groups was assessed using the χ^2^ test or Fisher. Bonferroni approach is taken in the subgroup analysis. Second, to further explore the association between food group consumption and perceived stress level, a logistic regression model and stepwise method were applied while controlling for basic characteristics, such as sex, age, and residence.

Stratified analyses were conducted by sex (female vs. male). Tests were 2-sided at the 0.05 significance level. All analyses were performed using SAS statistical software version 9.4 (SAS Institute Inc., Cary, NC, USA).

## Results

### Basic information

Of the 7,434 participants, 3,464 (46.6%) were males and 3970 (53.4%) were females. Most (51.1%) of the study participants were middle-aged people (aged 45–64 years). In addition, 23.7% were young adulthood (aged 19–44 years), and 25.1% were older adulthood (aged 65 years and older) [55]. The median of the PSS-14 total score was 25. People who perceived a higher level of stress (PSS-14 total score > median) made up 41.5% and 45.1% of the male and female groups, respectively (χ^2^ = 9.605, *p* = 0.002). Detailed information on the characteristics is shown in Table 1.

**Table 1:** Descriptive statistics of participants n (%) or median (IQR)

Variables		Male 3464 (46.6%)						Female 3970 (53.4%)				χ^2^	*P*
	Total	Higher stress 1439 (41.5%)	Lower stress 2025 (58.5%)	χ^2^	*P*		Total	Higher stress 1791 (45.1%)	Lower stress 2179 (54.9%)	χ^2^	*P*		
Age in 2011 wave, years	52.0(41.0–61.0)	52.0(41.0–61.0)	52.0(41.0–61.0)	-0.070	0.944		51.0(41.0–60.0)	52.0(41.0–61.0)	50.0(41.0–60.0)	1.693	0.091	1.773	0.076
Age in 2015 wave, years	56.0(45.0–65.0)	56.0(45.0–65.0)	56.0(45.0–65.0)	-0.052	0.959		55.0(45.0–64.0)	56.0(45.0–65.0)	54.0(45.0–64.0)	1.695	0.090	1.738	0.082
Marital status in 2011				4.563	0.207					4.186	0.242	107.189	< 0.001
Divorced/Separated/Widowed	174(5.0)	85(5.9)	89(4.4)				419(10.6)	204(11.4)	215(9.9)				
Married	3101(89.5)	1281(89.0)	1820(89.9)				3429(86.4)	1537(85.8)	1892(86.8)				
Never married	181(5.2)	70(4.9)	111(5.5)				105(2.6)	45(2.5)	60(2.8)				
Marital status in 2015				8.281	0.016					3.632	0.163	128.857	< 0.001
Divorced/Separated/Widowed	171(5.0)	89(6.2)	82(4.1)				463(11.7)	224(12.6)	239(11.0)				
Married	3165(91.6)	1295(90.3)	1870(92.5)				3431(86.7)	1537(86.1)	1894(87.2)				
Never married	120(3.5)	50(3.5)	70(3.5)				64(1.6)	24(1.3)	40(1.8)				
BMI in 2011	23.8(21.5–26.1)	23.6(21.3–26.0)	23.9(21.7–26.2)	-2.488	0.013		23.6(21.4–26.2)	23.59(21.4–26.0)	23.7(21.4–26.3)	-1.284	0.199	0.877	0.381
BMI categories in 2011				9.184	0.027					6.205	0.102	14.615	0.002
Normal weight	1643(49.0)	691(50.0)	952(48.3)				1912(49.0)	851(48.6)	1061(49.4)				
Obese	393(11.7)	153(11.1)	240(12.2)				529(13.6)	224(12.8)	305(14.2)				
Overweight	1190(35.5)	471(34.1)	719(36.5)				1267(32.5)	574(32.8)	693(32.2)				
Underweight	128(3.8)	67(4.9)	61(3.0)				192(4.9)	101(5.8)	91(4.2)				
BMI in 2015	24.2(21.8–26.6)	24.0(21.6–26.5)	24.3(22.0-26.6)	-1.721	0.085		24.1(21.8–26.5)	24.1(21.8–26.5)	24.0(21.8–26.5)	-0.234	0.815	0.312	0.755
BMI categories in 2015				8.589	0.035					2.789	0.425	3.132	0.372
Normal weight	1347(44.2)	552(44.2)	795(44.2)				1639(45.1)	725(44.5)	914(45.5)				
Obese	424(13.9)	165(13.2)	259(14.4)				544(15.0)	232(14.3)	312(15.5)				
Overweight	1150(37.7)	466(37.3)	684(38.1)				1304(35.9)	606(37.2)	698(34.8)				
Underweight	126(4.1)	67(5.4)	59(3.3)				149(4.1)	65(4.0)	84(4.2)				
Residence				12.726	< 0.001					44.844	< 0.001	0.077	0.782
Rural	2132(61.6)	936(65.1)	1196(59.1)				2431(61.2)	1199(66.95)	1232(56.5)				
Urban	1332(38.5)	503(35.0)	829(40.9)				1539(38.8)	592(33.05)	947(43.5)				
Urbanization index in 2011	73.2(54.6–88.9)	69.6(53.1–88.0)	75.7(56.2–89.5)	-4.859	< 0.001		73.8(54.7–88.9)	68.9(53.1–87.9)	78.2(56.2–90.0)	-6.547	< 0.001	-0.398	0.691
Weight in 2011, kg	66.5(59.3–75.0)	65.4(58.0-73.9)	67.3(60.0–75.0)	-4.138	< 0.001		58.0(51.9–65.0)	57.5(51.0-64.3)	58.3(52.2–65.0)	-3.641	< 0.001	32.011	< 0.001
Height in 2011, cm	168.0(162.8–172.0)	167.2(162.0-171.6)	168.0(163.2-172.1)	-3.774	< 0.001		156.5(152.0-160.2)	156.0(151.50–160.0)	157.0(152.7-160.6)	-5.069	< 0.001	56.622	< 0.001
Waist circumference in 2011, cm	86.0(79.3–93.0)	85.0(78.6–92.2)	87.0(80.0-93.1)	-2.913	0.004		82.0(75.0-89.6)	82.0(75.00–89.0)	82.0(75.0–90.0)	-0.805	0.421	15.561	< 0.001
Urbanization index in 2015	77.1(60.1–87.6)	73.6(57.5–87.0)	79.5(60.8–88.1)	-4.979	< 0.001		77.1(60.1–87.7)	73.3(58.1–86.8)	80.0(61.3–89.1)	-6.269	< 0.001	-0.503	0.615
Weight in 2015, kg	67.6(59.8–75.2)	66.9(58.7–75.0)	67.9(60.5–75.6)	-2.789	0.005		58.7(52.7–65.2)	58.4(52.0–65.0)	59.1(53.1–65.4)	-2.617	0.009	30.232	< 0.001
Height in 2015, cm	167.1(162.2-171.8)	166.5(161.7-171.5)	167.9(163.0-172.0)	-3.316	< 0.001		156.0(152.0-160.3)	155.5(151.0-160.0)	157.0(152.3-160.7)	-4.916	< 0.001	53.263	< 0.001
Waist circumference in 2015, cm	87.6(80.0–95.0)	87.0(80.0–94.0)	88.0(80.5–95.0)	-1.851	0.064		83.5(76.1–90.5)	83.2(76.0–90.0)	84.0(77.0–91.0)	-1.392	0.164	14.026	< 0.001
Cumulative average dietary intake													
Energy, kcal/day	2024.9(1558.2-2578.3)	2010.0(1553.3-2548.8)	2043.6(1562.0-2606.3)	-1.019	0.308		1692.1(1306.1-2147.5)	1688.6(1314.6-2172.7)	1693.3(1301.5-2132.1)	0.385	0.701	19.194	< 0.001
Protein, g/day	69.3(50.6–90.7)	67.6(49.3–88.5)	70.1(51.4–93.1)	-2.704	0.007		58.0(42.6–78.4)	56.6(42.5–76.5)	58.8(42.7–79.6)	-1.825	0.068	15.704	< 0.001
Carbohydrate, g/day	277.8(198.9-373.6)	279.4(197.8-374.5)	276.5(199.3-373.4)	0.035	0.972		238.5(171.9-314.5)	239.8(178.2-321.6)	237.4(167.6-310.6)	2.471	0.014	14.179	< 0.001
Fat, g/day	63.3(39.2–94.6)	62.1(37.6–91.6)	64.7(39.9–95.3)	-1.578	0.115		54.8(33.1–82.0)	53.5(32.1–80.4)	55.7(34.2–83.0)	-2.141	0.032	9.709	< 0.001
Calcium, mg/day	384.7(259.4-571.6)	373.6(251.0-545.1)	392.4(265.0-593.9)	-3.152	0.002		343.4(228.6-513.6)	328.3(221.0-487.4)	360.6(233.6-534.8)	-4.153	< 0.001	7.670	< 0.001
Sodium, mg/day	4056.2(2841.6-5646.4)	4041.5(2841.6-5608.4)	4074.6(2841.5-5665.7)	-0.485	0.628		3561.9(2518.3-4851.4)	3562.3(2540.9-4800.8)	3556.9(2508.1-4882.5)	0.102	0.919	9.881	< 0.001

### The distribution of dietary foods in male and female

The intake of food groups of male and female were presented in Table 2. Compared with male, female consumed higher proportions of vitamin A-rich fruits, citrus, other fruits (fruits not classified as citrus fruits or vitamin A-rich fruits), baked sweets, yogurt, nuts & seeds, and fluid milk (*p* < 0.05). Meanwhile, the level of unprocessed red meat (ruminant or nonruminant), poultry, sodas/sugar-sweetened beverages SSBs (sodas) consumption was higher in male (*p* < 0.05).

**Table 2:** Percentages of individuals (n (%)) in male and female who reported intake from food groups in 2011

Group code	Food groups	Total	Male	Female	χ^2^	*P*
1	Staple foods made from grain	7401(99.6)	3446(99.5)	3955(99.6)	0.842	0.359
2	Whole grains	1611(21.7)	717(20.7)	894(22.5)	3.611	0.057
3	White roots/tubers	2390(32.2)	1096(31.6)	1294(32.6)	0.773	0.379
4	Legumes	3154(42.4)	1475(42.6)	1679(42.3)	0.063	0.802
5	Vitamin A-rich orange vegetables	949(12.8)	420(12.1)	529(13.3)	2.393	0.122
6	Dark green leafy vegetables	4304(57.9)	2020(58.3)	2284(57.5)	0.465	0.495
7	Other vegetables	6284(84.5)	2926(84.5)	3358(84.6)	0.019	0.891
**8**	**Vitamin A-rich fruits**	**154(2.1)**	**57(1.7)**	**97(2.4)**	**5.804**	**0.016**
**9**	**Citrus**	**494(6.7)**	**186(5.4)**	**308(7.8)**	**17.01**	**< 0.001**
**10**	**Other fruits**	**2435(32.8)**	**1031(29.8)**	**1404(35.4)**	**26.36**	**< 0.001**
**11**	**Baked sweets**	**812(10.9)**	**344(9.9)**	**468(11.8)**	**6.561**	**0.010**
12	Other sweets	84(1.1)	40(1.2)	44(1.1)	0.036	0.850
13	Eggs	2973(40.0)	1392(40.2)	1581(39.8)	0.101	0.751
14	Cheese	6(0.1)	2(0.1)	4(0.1)	-	0.856
**15**	**Yogurt**	**217(2.9)**	**81(2.3)**	**136(3.4)**	**7.718**	**0.006**
16	Processed meat	272(3.7)	133(3.8)	139(3.5)	0.600	0.438
**17**	**Unprocessed red meat (ruminant)**	**591(8.0)**	**310(9.0)**	**281(7.1)**	**8.850**	**0.003**
**18**	**Unprocessed red meat (nonruminant)**	**4526(60.9)**	**2176(62.8)**	**2350(59.2)**	**10.199**	**0.001**
**19**	**Poultry**	**1076(14.5)**	**538(15.5)**	**538(13.6)**	**5.856**	**0.016**
20	Fish & seafood	1732(23.3)	821(23.7)	911(23.0)	0.588	0.443
**21**	**Nuts & seeds**	**696(9.4)**	**276(8.0)**	**420(10.6)**	**14.87**	**< 0.001**
22	Ultra-processed packaged salty snacks	17(0.2)	7(0.2)	10(0.3)	0.201	0.654
23	Instant noodles	93(1.3)	43(1.2)	50(1.3)	0.005	0.944
24	Deep fried foods	569(7.7)	282(8.1)	287(7.2)	2.175	0.140
**25**	**Fluid milk**	**711(9.6)**	**306(8.8)**	**405(10.2)**	**4.001**	**0.046**
26	Sweetened tea/coffee/milk drinks	74(1.0)	32(0.9)	42(1.1)	0.338	0.561
27	Fruit juice	55(0.7)	24(0.7)	31(0.8)	0.195	0.659
**28**	**Sodas/sugar-sweetened beverages SSBs (sodas)**	**43(0.6)**	**30(0.9)**	**13(0.3)**	**9.331**	**0.002**
29	Fast food	32(0.4)	15(0.4)	17(0.4)	0.001	0.975

### The distribution of dietary foods in different stress groups

The percentage of individuals reporting consumption of the 29 food groups in the two stress groups are presented in Table 3. Compared with the higher-stress group, the lower-stress group had higher proportions of legumes, other vegetables (vegetables not classified as white roots/tubers, legumes, vitamin A-rich orange vegetables, or dark green leafy vegetables), other fruits, baked sweets, eggs, yogurt, unprocessed red meat (nonruminant), poultry, fish & seafood, nuts & seeds, fluid milk, and fruit juice (*p* < 0.05). Meanwhile, the level of Vitamin A-rich orange vegetables consumption was higher in the higher-stress group (*p* = 0.032) compared to the lower-stress group.

**Table 3:** Percentages of individuals (n (%)) in lower and higher stress groups who reported intake from food groups in 2011

Group code	Food groups	Higher stress (*n* = 3230)	Lower stress (*n* = 4204)	χ^2^	*P*
1	Staple foods made from grain	3213(99.5)	4188(99.6)	0.878	0.349
2	Whole grains	695(21.5)	916(21.8)	0.080	0.778
3	White roots/tubers	1033(32.0)	1357(32.3)	0.074	0.786
**4**	**Legumes**	**1318(40.8)**	**1836(43.7)**	**6.150**	**0.013**
**5**	**Vitamin A-rich orange vegetables**	**443(13.7)**	**506(12.0)**	**4.624**	**0.032**
6	Dark green leafy vegetables	1875(58.1)	2429(57.8)	0.055	0.814
**7**	**Other vegetables**	**2692(83.3)**	**3592(85.4)**	**6.153**	**0.013**
8	Vitamin A-rich fruits	73(2.3)	81(1.9)	1.000	0.317
9	Citrus	200(6.2)	294(7.0)	1.891	0.169
**10**	**Other fruits**	**981(30.4)**	**1454(34.6)**	**14.73**	< 0.001
**11**	**Baked sweets**	**316(9.8)**	**496(11.8)**	**7.623**	**0.006**
**12**	**Other sweets**	**28(0.9)**	**56(1.3)**	**3.538**	**0.060**
**13**	**Eggs**	**1238(38.3)**	**1735(41.3)**	**6.588**	**0.010**
14	Cheese	0(0.0)	6(0.1)	-	0.856
**15**	**Yogurt**	**68(2.1)**	**149(3.5)**	**13.35**	**< 0.001**
16	Processed meat	106(3.3)	166(4.0)	2.305	0.129
17	Unprocessed red meat (ruminant)	241(7.5)	350(8.3)	1.864	0.172
**18**	**Unprocessed red meat (nonruminant)**	**1919(59.4)**	**2607(62.0)**	**5.187**	**0.023**
**19**	**Poultry**	**426(13.2)**	**650(15.5)**	**7.621**	**0.006**
**20**	**Fish & seafood**	**700(21.7)**	**1032(24.6)**	**8.456**	**0.004**
**21**	**Nuts & seeds**	**268(8.3)**	**428(10.2)**	**7.637**	**0.006**
22	Ultra-processed packaged salty snacks	10(0.3)	7(0.2)	1.639	0.200
23	Instant noodles	40(1.2)	53(1.3)	0.007	0.932
24	Deep fried foods	231(7.2)	338(8.0)	2.039	0.153
**25**	**Fluid milk**	**251(7.8)**	**460(10.9)**	**21.24**	**< 0.001**
26	Sweetened tea/coffee/milk drinks	32(1.0)	42(1.0)	0.001	0.971
**27**	**Fruit juice**	**15(0.5)**	**40(1.0)**	**5.901**	**0.015**
28	Sodas/sugar-sweetened beverages SSBs (sodas)	14(0.4)	29(0.7)	2.088	0.149
29	Fast food	12(0.4)	20(0.5)	0.463	0.496

### The relationship between food groups and perceived stress level

Unconditional multivariate logistic regression demonstrated that increased intake of food groups such as legumes, other vegetables, other fruits, yogurt, poultry, fish & seafood, fluid milk, and fruit juice correlates with less perceived stress (OR range: 0.544–0.892). Detailed information is shown in Table 4.

**Table 4:** Associations of food groups in 2011 with perceived stress level in 2015

Variables	Estimate	Standard error	χ^2^	*p*	OR	95%CI
Intercept	0.048	0.066	0.518	0.472		
Sex	-0.080	0.024	11.543	**0.001**	0.851	0.776–0.934
Legumes	-0.118	0.048	6.129	**0.013**	0.889	0.809–0.976
Other vegetables	-0.150	0.065	5.380	**0.020**	0.860	0.758–0.977
Other fruits	-0.149	0.051	8.436	**0.004**	0.862	0.779–0.953
Yogurt	-0.454	0.150	9.188	**0.002**	0.635	0.473–0.852
Poultry	-0.139	0.068	4.182	**0.041**	0.870	0.762–0.994
Fish & seafood	-0.114	0.057	4.066	**0.044**	0.892	0.799–0.997
Fluid milk	-0.315	0.084	14.076	**< 0.001**	0.730	0.619–0.860
Fruit juice	-0.609	0.306	3.959	**0.047**	0.544	0.299–0.991

*Note* Other vegetables indicate vegetables not classified as white roots/tubers, legumes, vitamin A-rich orange vegetables, or dark green leafy vegetables. Other fruits indicate fruits not classified as citrus fruits or vitamin A-rich fruits. A logistic regression model and stepwise method were applied while controlling for basic characteristics, such as sex, age, and residence

### Stratified analysis by sex

**Fig. 1:**
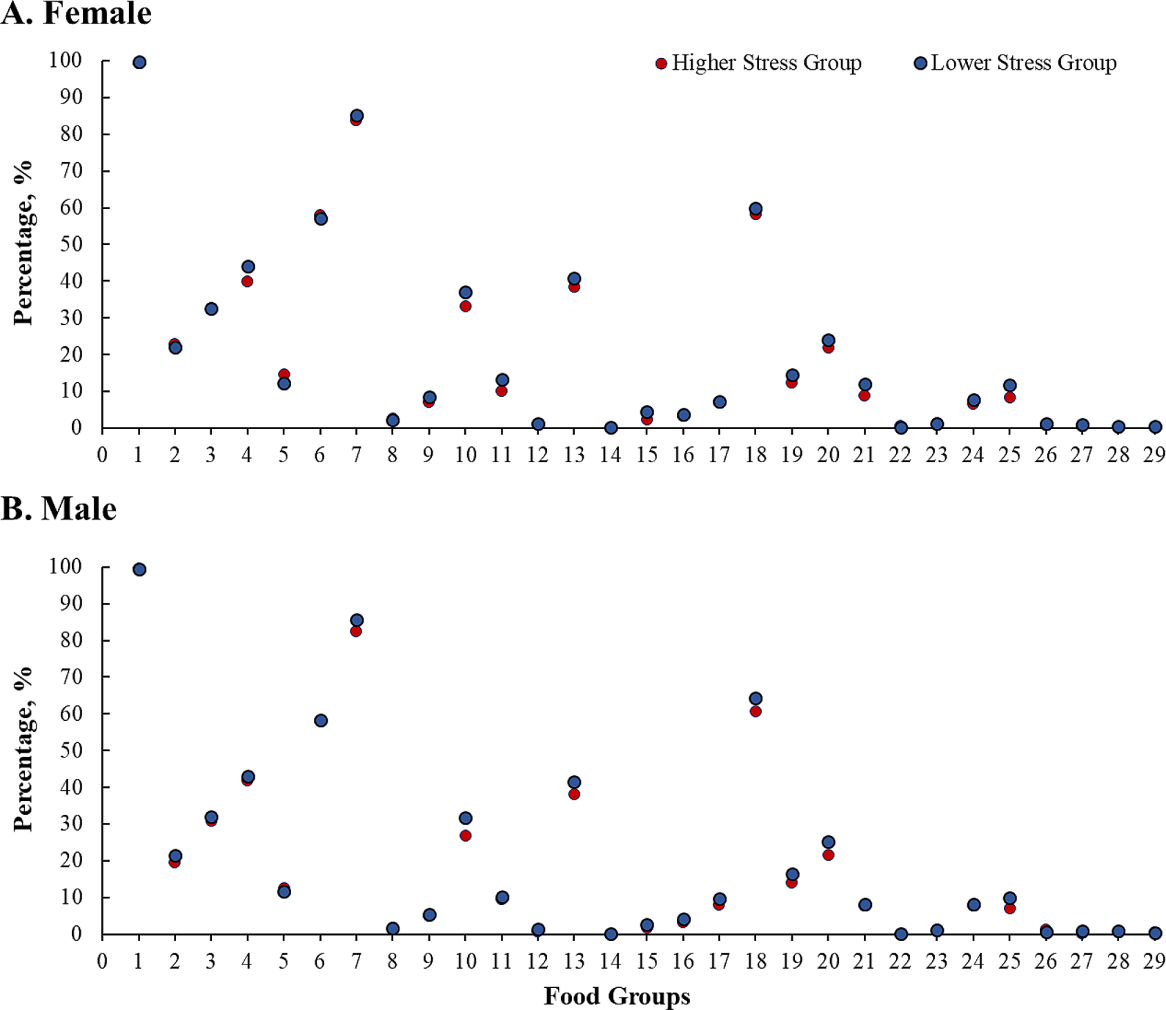
Sex-specific food groups in the higher stress group and lower stress group. *Note* Food group 1: staple foods made from grains; 2: whole grains; 3: white root/tubers; 4: legumes; 5: vitamin A-rich orange vegetables; 6: dark green leafy vegetables; 7: other vegetables; 8: vitamin A-rich fruits; 9: citrus; 10: other fruits; 11: grain-based sweets; 12: other sweets; 13: eggs; 14: cheese; 15: yogurt; 16: processed meats; 17: unprocessed red meat (ruminant); 18: unprocessed red meat (nonruminant); 19: poultry; 20: fish & seafood; 21: nuts & seeds; 22: packaged ultra-processed salty snacks; 23: instant noodles; 24: deep fried foods; 25: fluid milk; 26: sweetened tea/coffee/milk drinks; 27: fruit juice; 28: sugar-sweetened beverages (SSBs) (sodas); 29: fast food

In the stratified analyses for females and males (Fig. 1), there was a statistically significant difference in the proportion of other vegetables, other fruits, fish and seafood, fluid milk, and fruit juice between the two stress groups in men (*p* < 0.025). In the female group, the distribution of legumes, other fruits, baked sweets, yogurt, nuts and seeds and fluid milk between the two stress groups was statistically significant (*p* < 0.025).

## Discussion

In this national prospective study, we assessed relatively comprehensive food groups in adults using the DQQ and found significant associations between lower perceived stress and certain food groups, including legumes, other vegetables (vegetables not classified as white roots/tubers, legumes, vitamin A-rich orange vegetables, or dark green leafy vegetables), other fruits(fruits not classified as citrus fruits or vitamin A-rich fruits), yogurt, poultry, fish & seafood, fluid milk, and fruit juice. We also conducted stratified analysis by sex, which is a significant factor for perceived stress. In particular, vegetables, fruits, fish & seafood, and fruit juice were negatively associated with higher stress in men. Legumes, baked sweets, yogurt, and nuts & seeds were negatively associated with higher stress in women.

Increased intake of fresh fruits and vegetables may have psychological benefits [56]; for example, people with higher fruit and vegetable consumption in daily life are less depressed [57] and happier [58]. Moreover, Ghadeer S. Aljuraiban conducted a cross-sectional study in Saudi Arabia, which included 401 female college students aged 19–35 years. This study reported that healthy plant-based diets were associated with lower stress in young Saudi women [59]. The mechanisms were speculated to be that various antioxidants and anti-inflammatory components, such as fiber [60], polyphenols [61], magnesium [62], zinc, selenium, vitamin C, B vitamins [63], and carbohydrates in fruits and vegetables, were associated with increased dopaminergic synthesis and serotonin release [64]; this would lead promotion of positive emotion and incentive motivation [65]. Regarding dairy products, a wide range of dietary yogurts and milk currently contain probiotic bacteria [66], which have been suggested to favorably alter the gut microbiota and gut function [67] and improve psychological health [68]. Indeed, evidence suggests that dysfunction of the microbiome-gut-brain axis might be implicated in stress related disorders such as depression through regulation of tryptophan metabolism [57].

We found the consumption of poultry, rather than other types of meat, was negatively associated with higher perceived stress. A systematic review showed that those who avoided meat consumption had a significantly higher risk of depression [69]. A recent meta-analysis of observational studies also indicated that adherence to the vegetarian diet may increase the risk of depression [70]. However, some studies have suggested the opposite conclusion, meat consumption may be associated with a higher risk of depression and stress [71, 72]. A potential source of variation in the meat-stress association may be meat types [73]. “Red meat,” especially high fat meat, was proven to impact the response to stress and promote depressive- and anxiety-like behaviors [74], by suppressing hypothalamic protein kinase A (PKA) signaling [75]. Poultry, classified as “white meat,” contains moderate energy, highly digestible proteins of good nutritional quality, unsaturated lipids, B-group vitamins, and minerals (such as iron, zinc, and copper) [76]. An investigation in an Iranian population found that white meat intake was inversely associated with psychological distress symptoms [73]. A study conducted in Finland also showed that subjects with a lower risk for stress-related disorders consumed white meat more often [77].

Fish and seafood are universally known food groups that are good for physical and psychological health [78]. Numerous studies have revealed that fish, as the primary dietary sources of eicosapentaenoic acid (EPA) and docosahexaenoic acid (DHA), is associated with a reduced risk of depression [79, 80]. A meta-analysis involving 26 studies indicated that high fish consumption can reduce the risk of depression [81]. The postulated mechanisms of EPA and DHA’s anti-depressive effects were demonstrated, such as reducing the occurrence of inflammation, decreasing the production of arachidonic acid [82], and enhancing the production of neuroprotective metabolites [80]. A nationwide longitudinal study conducted in Japan indicated that women with higher fish and/or n-3 PUFA intake showed a reduced risk of postpartum depression [34]. The present study adds important information to the field in the Chinese population. Regarding interventional studies, a randomized, placebo-controlled trial in abstinent alcoholics demonstrated that fish oil supplementation reduces cortisol basal levels and perceived stress [83].

Our result was consistent with the current evidence that sex-specific associations were observed between food groups and perceived stress. For example, a prospective study found that the intake of yogurt is related to a lower risk of depression among women but not men [84]. Western dietary patterns and high levels of triglycerides have been found to be associated with cognitive impairment in men but not in women [41]. Western pattern is usually rich in calories, saturated/trans-unsaturated fatty acids, sugar, and alcohol, which are associated with cognitive decline [85]. Moreover, the potential mechanistic links between Western diet consumption and cognition is that the Western diet includes heat-processed foods that contain high levels of advanced glycation end products (AGEs). Elevated levels of AGEs were associated with increased aggregation and cytotoxicity of amyloid-β (Aβ) [86]. Animal studies demonstrated that increased levels of circulating plasma Aβ could cause blood-brain barrier degradation and hippocampal dysfunction, and these condition are associated with cognitive impairment [87]. Eating fruit was found to be associated with increased positive affect among men but not women [65]. In addition, special attention should be given to the fact that intake of vitamin A-rich orange vegetables was higher in higher stress group of women. This finding was in line with recent reviews, which reported that elevated retinoid levels form a significant risk factor for depressive symptoms [88]. These sex-specific associations can be explained by sex-specific differences in dietary intake [89]. Moreover, sex-based differences in biophysiological sensitivity, such as sex hormones and inflammation, to dietary intake may further explain the observed association between various food groups and psychological health [60]. In addition, their brain morphology and connectivity are different, and the dimorphic state of the brain may also influence nutritional needs, behavioral traits as well as susceptibility to pressure [90].

In summary, these results were consistent with previous findings; for example, a traditional whole-food diet, consisting of higher intake of foods such as vegetables, fruits, seafood, whole grains, poultry meat, nuts, and legumes, with avoidance of processed foods, was more likely to provide the nutrients that contribute to the prevention of this stress-related disorder [91]. Similarly, the Mediterranean diet, which is high in fruits, vegetables, wholegrains, legumes, seafood, nuts, seeds, and olive oil, was indicated to be of significant benefit for psychological health [31]. Better overall diet quality is proven to be associated with a lower risk for stress and stress-associated disorders [92]. On the other hand, intake of nutritional supplementation could also affect stress and psychological status. For example, the meta-analyses by Mikola [93] and Dominika [94]concluded that vitamin D supplementation ≥ 2,000 IU/day may effectively alleviate the symptoms of depression in children and adults. As neurosteroid and immunological actions of vitamin D may regulate depression-linked physiology. The meta-analyses by Lauren provided evidence for the benefit of B vitamin supplementation in healthy and at-risk populations for stress [95]. The results from a cross‑sectional analysis and a randomized controlled trial indicated that Vitamin C supplementation promotes mental vitality in healthy young adults, and Vitamin C may has modulating effects on neurotransmitters and hormones in the brain [96]. The post-hoc analysis of a randomized controlled trial found that magnesium and vitamin B6 supplementation could provide a meaningful clinical benefit in daily life for individuals with stress [97].

### Strengths and limitations

In this study, we examined the relationship between 29 food groups measured using the China-adapted DQQ and perceived stress, prospectively. First, legumes, vegetables, fruits, yogurt, poultry, fish& seafood, fluid milk, and fruit juice were associated with lower perceived stress in general adults. This finding provides new information by contributing to the growing body of literature examining the association between food groups and perceived stress. Second, the current study conducted sex-based analyses in this area of research with the assumption that the benefits of a particular food group may unequally contribute to the perceived stress of men and women. The analysis supports the need for sex-based analyses to better understand the association between dietary intake and stress. Third, the CHNS data used in this study was obtained from a national representative sample, which provides a basis for monitoring the reliability of its findings.

There were, however, also some limitations. First, this study is essentially descriptive research, and its ability to make valid causal inferences was limited. Future replication and investigation are needed, such as animal studies and randomized controlled trials. Second, a four-year gap existed between dietary information and stress assessment. During this period, a person’s dietary habits can remain stable or vary with changing circumstances. Although our result can explain the order of occurrence of diet and PSS, the diet in weeks before PSS evaluation in 2015 was not analyzed, this would be a limitation. While our design was in line with previous studies [11, 98, 99]. For example, Zhang, J., & Zhao, A. explored the effects of dietary diversity score (DDS) on healthy aging using the data of CHNS. In their study, DDSs were calculated using the dietary data collected in the years 2009 and 2011, and the healthy aging score (HAS) was calculated based on the data collected in the year 2015 [99]. Moreover, previous studies indicated that eating habits of participant may not change significantly during 2011 and 2015. According to the study of Song et al., [100], the mean total energy intake of participants in CHNS was 2091.51(716.06) kcal/day and 2009.22 (717.43) kcal/day, respectively. Another Results from the CHNS also indicated that the percentage of energy from protein, fat, carbohydrate were 32.6%, 54.4%, and 12.7% in 2011, 35.6%, 51.6%, and 12.6% in 2015, respectively [101]. To some extent, these evidences suggested that the participants’ dietary habits and lifestyle choices were relatively stable. Third, dietary data and stress levels were assessed through self-report, which is subjective and may be subject to recall bias.

## Conclusions

In conclusion, food groups measured using the DQQ for China were differentially associated with perceived stress in this prospective analysis of a national population. Higher intake of certain food groups such as other vegetables, other fruits, dairy products, seafood, poultry meat, and legumes was associated with lower perceived stress.

### Electronic supplementary material

Below is the link to the electronic supplementary material.

Supplementary Material 1

## Data Availability

The dataset in the present study was open-accessed and freely obtained from the CHNS website with registration at https://www.cpc.unc.edu/projects/china/data/datasets/ (accessed on 22 March 2021).
