# Supplementary material for: Association between 29 food groups of diet quality questionnaire and perceived stress in Chinese adults: a prospective study from China health and nutrition survey
Source: BMC Public Health. 2024 Jul 9;24:1832. doi: 10.1186/s12889-024-19308-w (PMC11234725; doi:10.1186/s12889-024-19308-w)
Supplement: Supplementary file 1 — Supplementary Material 1 [file 12889_2024_19308_MOESM1_ESM.docx]

Supplementary material

Table S1 The contents of 14-item perceived stress scale

| Item number | Item content |
| --- | --- |
| 1 | felt upset because of something that happened unexpectedly? |
| 2 | felt like you couldn't control the important things in your life? |
| 3 | felt nervous or stressed? |
| 4 | dealt well with life hassles? |
| 5 | coped well with important changes in your life? |
| 6 | felt able to handle your personal problems? |
| 7 | felt things were going your way? |
| 8 | felt unable to cope with all the things that you had to do? |
| 9 | felt able to control irritations in your life? |
| 10 | felt you were on top of things? |
| 11 | felt angered because of things that happened outside of your control? |
| 12 | found yourself thinking about all the things that you have to do? |
| 13 | felt able to control how you spend your time? |
| 14 | felt troubles were piling up so high that you could not deal with them? |

*Note. Every item started with the sentence “In the last month, how often have you….. ". The questions were examined on a 5-point Likert-type scale, ranging from 0 = “never” to 4 = “very often”.*
